# Single neonatal irradiation induces long-term gene expression changes in the thyroid gland, which may be involved in the tumorigenesis

**DOI:** 10.1038/s41598-021-03012-5

**Published:** 2021-12-08

**Authors:** Nariaki Fujimoto, Mutsumi Matsuu-Matsuyama, Masahiro Nakashima

**Affiliations:** 1 Research Institute for Radiation Biology and Medicine (RIRBM), Hiroshima University, Hiroshima, Japan; 2 Tissue and Histopathology Section, Atomic Bomb Disease Institute, Nagasaki University, Nagasaki, Japan; 3 Department of Tumor and Diagnostic Pathology, Atomic Bomb Disease Institute, Nagasaki University, Nagasaki, Japan

**Keywords:** Endocrinology, Oncogenesis, Endocrine cancer, Paediatric cancer, Tumour biomarkers

## Abstract

Exposure to ionizing radiation in childhood has been recognized as a risk factor for thyroid cancer. We previously demonstrated that neonatal X-irradiation induced specific deformation of the thyroid follicles. Here, we further analyzed this model to understand the possible relationship with thyroid carcinogenesis. Wistar rats were subjected to cervical X-irradiation at different ages of 1–8 weeks old and at different doses of 1.5–12 Gy. For tumor promotion, rats were fed with an iodine-deficient diet (IDD). In cervically X-irradiated neonatal rats, the size of thyroid follicles decreased, accompanied by an increase in the number of TUNEL-positive cells. *Fas* and *Lgals3* mRNA levels increased, while *Mct8* and *Lat4* expressions decreased. The co-administration of IDD induced the proliferation and the upregulation in *Lgals3* expression, resulting in thyroid adenoma development at 28 weeks post-exposure. Our data demonstrated that single neonatal X-irradiation induced continuous apoptotic activity in the thyroid with the long-term alternation in *Fas*, *Mct8*, *Lat4*, and *Lgals3* mRNA expressions. Some of these changes were similar to those induced by IDD, suggesting that neonatal X-irradiation may partially act as a thyroid tumor promoter. These radiation-induced thyroidal changes may be enhanced by the combined treatment with IDD, resulting in the early development of thyroid adenoma.

## Introduction

Childhood and adolescence radiation exposure is associated with thyroid carcinogenesis^1–3^. Studies of the infants treated with x-rays before the 1950s already indicate an association^4^. The cohort of Japanese atomic bomb survivors also indicated an excess thyroid cancer risk linked with childhood radiation exposure^5^. The risk was re-recognized by a rapid rise in childhood thyroid cancer cases after the Chernobyl nuclear plant accident^6,7^. The typical type is papillary thyroid cancer, which emerges 5–10 years after irradiation^8^. Studies of sporadic papillary thyroid cancer have shown that two oncogenic changes, *RET* rearrangement and *BRAF* point mutations, were most frequently involved in thyroid carcinogenesis^9^. Although similar changes in RET and BRAF were found in the post-Chernobyl thyroid cancer cases, they are not associated with the radiation exposure but strongly related to the patient's age at operation^3^. In addition to these genomic alterations, the changes in gene expressions are related to thyroid carcinogenesis^10^.

Although human cases have been investigated extensively, the underlying mechanisms of childhood's susceptibility to thyroid carcinogenesis are yet to be elucidated. A high mitotic rate of thyroid follicular cells in infancy has been suggested to be responsible for the high prevalence of thyroid cancer^11,12^. An early study in rats exposed to cervical X-irradiation showed that neonatal rats were indeed more susceptible to developing thyroid cancer than adult animals^13^. However, studies to investigate the underlying mechanisms in animal models have rarely been performed^14^. Previously, we cervically X-irradiated 5–6 days old Wistar rats and, after comparing them with rats irradiated at 8 weeks old, found that thyroid follicle sizes were reduced only after the neonatal irradiation^15^. In the present study, we further investigated this animal model by examining the effects at different radiation doses and ages. Since the thyroid doses among the children in the Chernobyl accident were mainly less than 3 Gy^16^, the effects of 1.5 and 3 Gy of X-irradiation were investigated in addition to previously examined 6 and 12 Gy. In order to find the possible relationship between the initial thyroidal changes caused by neonatal-irradiation and thyroid tumor development, mRNA expressions of thyroid cancer-related marker genes, *Mct8*, *Lat4*, *Met*, and *Lgals3*, were determined. We also examined these parameters in the thyroid tumors induced by neonatal irradiation plus feeding an iodine-deficient diet (IDD).

## Results

### Body and thyroid weights

Body and thyroid weights at the necropsy were recorded and summarized in Table 1. Increases in body weight were steady in all groups of every experiment, although the increasing rates were significantly reduced by neonatal irradiation or feeding with IDD. In Experiment 1, where rats were examined at the age of 9 weeks, the average body weights were significantly lower in the 1w12Gy group than that in the Control. In Experiment 2, where rats were necropsied at the age of 17 weeks, the average body weight was significantly reduced only in the 1w12Gy group. In Experiment 3, which ended 28 weeks after the irradiation, the average body weights in IDD and 1w12Gy-IDD were 43% and 37% of those in the Control, respectively. The absolute thyroid weights also decreased along with reduced body weights, but the relative thyroid weights were not significantly affected in Experiments 1 or 2. Feeding with IDD significantly increased the absolute and relative thyroid weights in IDD and 1w12Gy-IDD groups by about 31 and 21 times than those in the Control, respectively.

**Table 1:** Body and thyroid weights.

Group	Treatment	Age of necropsy	Body weight (g)	Thyroid weight (mg)	Thyroid weight (mg/kg b.w.)
**Experiment 1**					
Control	ShamX at 5–6 days old	9 w.o	380 ± 8.9	18 ± 1.8	48 ± 4.7
1w1.5 Gy	1.5 Gy at 5–6 days old	9 w.o	390 ± 10.2	19 ± 1.3	49 ± 3.2
1w3Gy	3 Gy at 5–6 days old	9 w.o	355 ± 7.8	17 ± 1.5	48 ± 5.0
1w6Gy	6 Gy at 5–6 days old	9 w.o	330 ± 19.9	14 ± 1.5	42 ± 1.9
1w12Gy	12 Gy at 5–6 days old	9 w.o	291 ± 22.9*	12 ± 1.0*	43 ± 3.4
**Experiment 2**					
Control	ShamX at 5–6 days old	17 w.o	607 ± 19.3	29 ± 2.2	48 ± 4.0
1w12Gy	12 Gy at 5–6 days old	17 w.o	446 ± 38.2*	15 ± 1.9**	34 ± 5.3
2w12Gy	12 Gy at 2 weeks old	17 w.o	557 ± 23.5	24 ± 2.1	42 ± 1.9
4w12Gy	12 Gy at 4 weeks old	17 w.o	576 ± 38.8	21 ± 0.5	37 ± 2.3
8w12Gy	12 Gy at 8 weeks old	17 w.o	541 ± 11.0	24 ± 1.6	45 ± 3.0
**Experiment 3**					
Control	ShamX at 5–6 days old	29 w.o	692 ± 11.5	21 ± 3.9	30 ± 5.5
1w12Gy	12 Gy at 5–6 days old	29 w.o	522 ± 42.6	15 ± 1.3	29 ± 3.9
IDD	IDD	29 w.o	298 ± 6.9**	293 ± 29**	993 ± 113**
1w12Gy-IDD	12 Gy at 5–6 days old + IDD	29 w.o	249 ± 15.6**	147 ± 21**	592 ± 65**

The numbers of rats in each group were 6 rats/group in Experiment 1; 5 rats/group in Experiment 2; 4, 3, 7, and 8 rats in Control, 1w12Gy, IDD, and 1w12Gy-IDD, respectively, in Experiment 3.

*, and ** indicate significant differences from each control (*p < 0.05; **p < 0.01).

### Changes in thyroid follicle size in cervically X-irradiated rats at different doses and different ages

**Figure 1:**
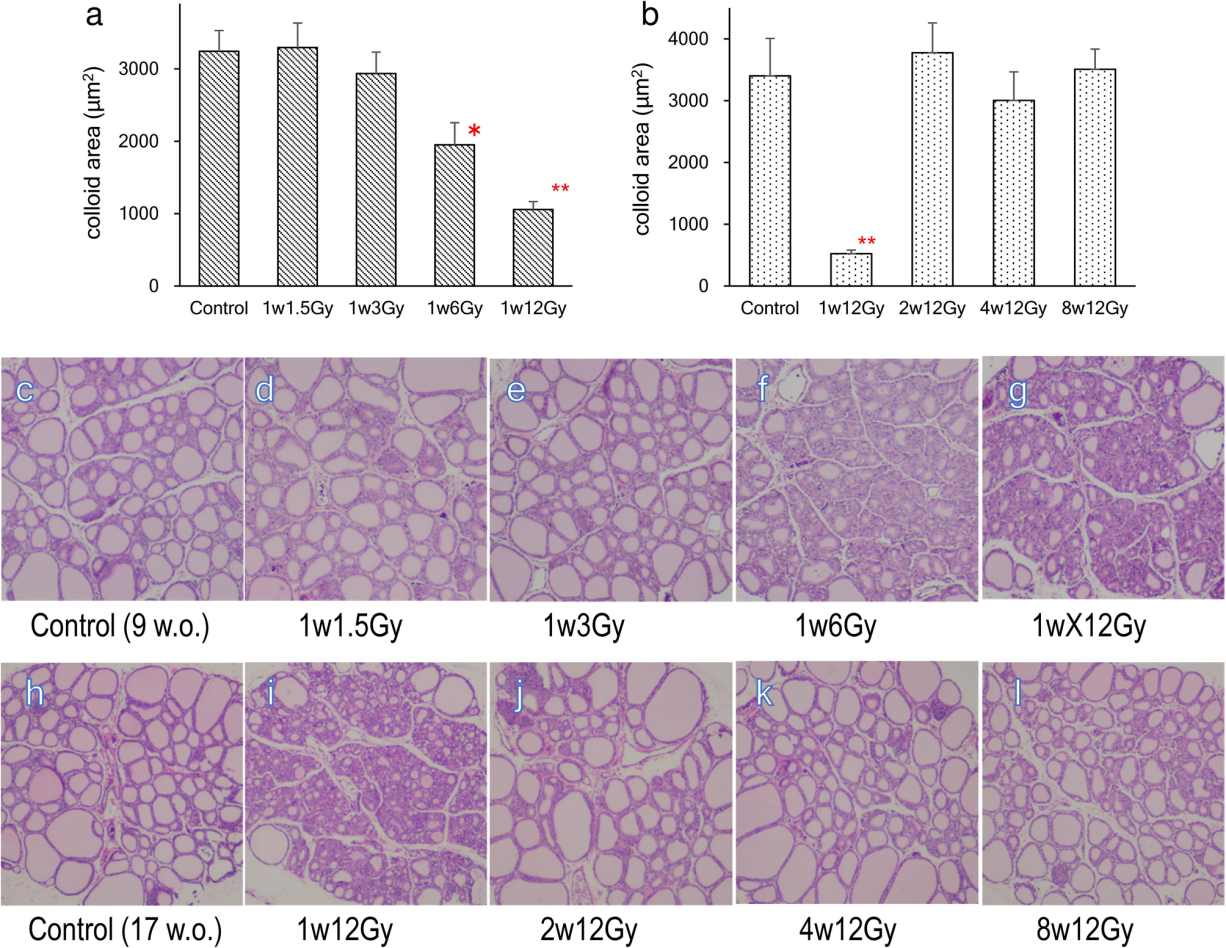
Morphological changes in the thyroid of rats cervically X-irradiated. The average colloid sizes in 9-week-old rats neonatally X-irradiated at doses of 0, 1.5, 3, 6, and 12 Gy (**a**) and in 17-week-old rats exposed to 12 Gy of X-rays at 1, 2, 4, and 8 weeks old (**b**). The follicular sizes were decreased by the x-irradiation at 6 Gy and 12 Gy (**c–g**). The reduction was not observed in rats irradiated at 2 weeks old or later (**h**–**l**). *, ** indicate significant difference from Control (*p < 0.05, **p < 0.01).

Figure 1 shows HE staining of the thyroid in cervically X-irradiated rats. In Experiment 1, the thyroid follicle sizes were reduced in irradiated groups in a dose-dependent manner (Fig. 1a and c–g). In Experiment 2, a significant reduction in the thyroid follicle size was observed only in the 1w12Gy group (Fig. 1b and h–l).

### Changes in numbers of TUNEL-positive and Ki-67 positive cells

**Figure 2:**
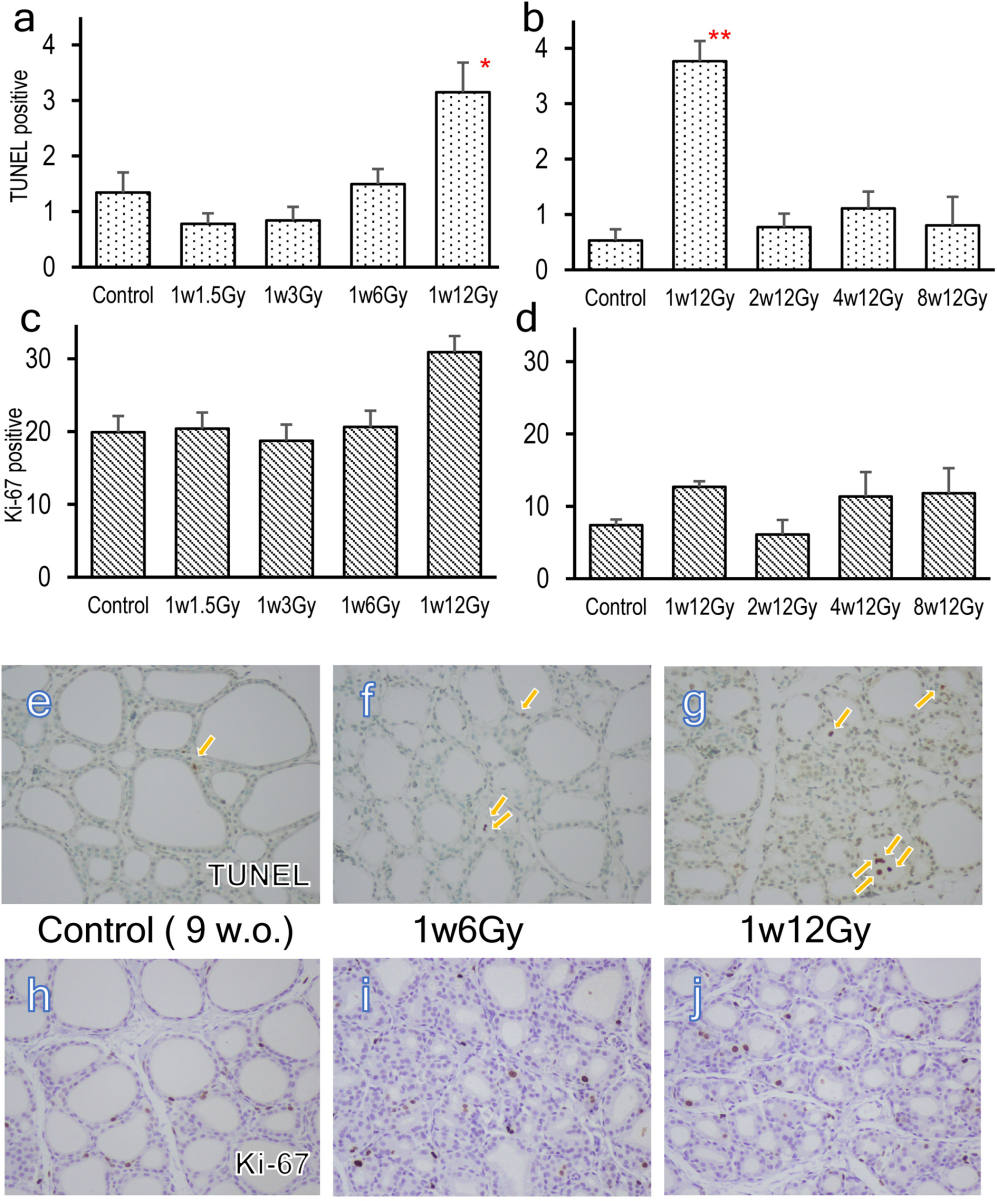
TUNEL and Ki-67 staining in the thyroid of the cervically X-irradiated rats. The numbers of TUNEL or Ki-67 positive cells in the thyroid of 9-week-old rats neonatally X-irradiated old at doses of 0, 1.5, 3, 6, and 12 Gy (**a**, **c**) and of 17 weeks-old rats exposed to 12 Gy of X-rays at 1, 2, 4, and 8 weeks old (b, d). Representative TUNEL (**e–g**) and Ki-67 (**h–j**) staining of Control (**e**, **h**), 1xW-6 Gy (**f**, **i**), and 1xW-12 Gy (**g**, **j**). Arrows indicate TUNEL positive staining. ** indicates significant difference from Control (*p < 0.05, **p < 0.01).

The results of TUNEL and Ki-67 staining are summarized in Fig. 2. In Experiment 1, the average number of TUNEL-positive cells was significantly increased only in the highest dose-treated group, 1w12Gy (Fig. 2a). In Experiment 2, the number of TUNEL-positive cells was elevated only in the neonatally X-irradiated group, 1w12Gy (Fig. 2b). TUNEL-positive cells were frequently observed inside the colloid area (Fig. 2g). On the other hand, the numbers of Ki-67 positive cells were not significantly altered after exposure to different doses of radiation (Fig. 2c) or by different ages of exposure (Fig. 2d).

### The neonatally X-irradiated thyroid in rats fed with IDD for 28 weeks

**Figure 3:**
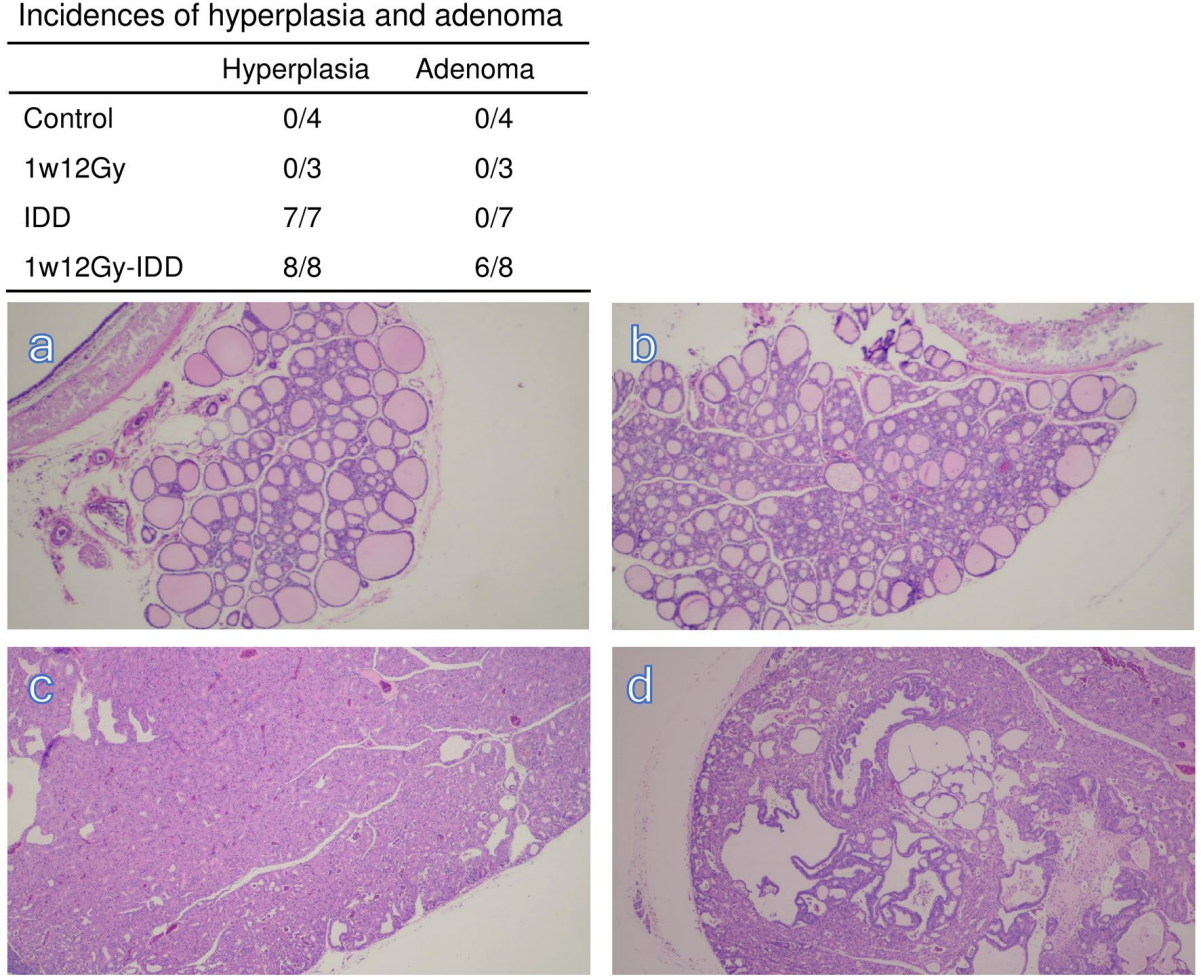
Thyroid tumors developed in neonatally X-irradiated rats fed with IDD at 29 weeks old. Representative H.E. staining of the thyroid in Control (**a**), 1w12Gy (**b**), IDD, diffused follicular hyperplasia (**c**), and 1w12Gy-IDD, follicular adenoma (**d**).

**Figure 4:**
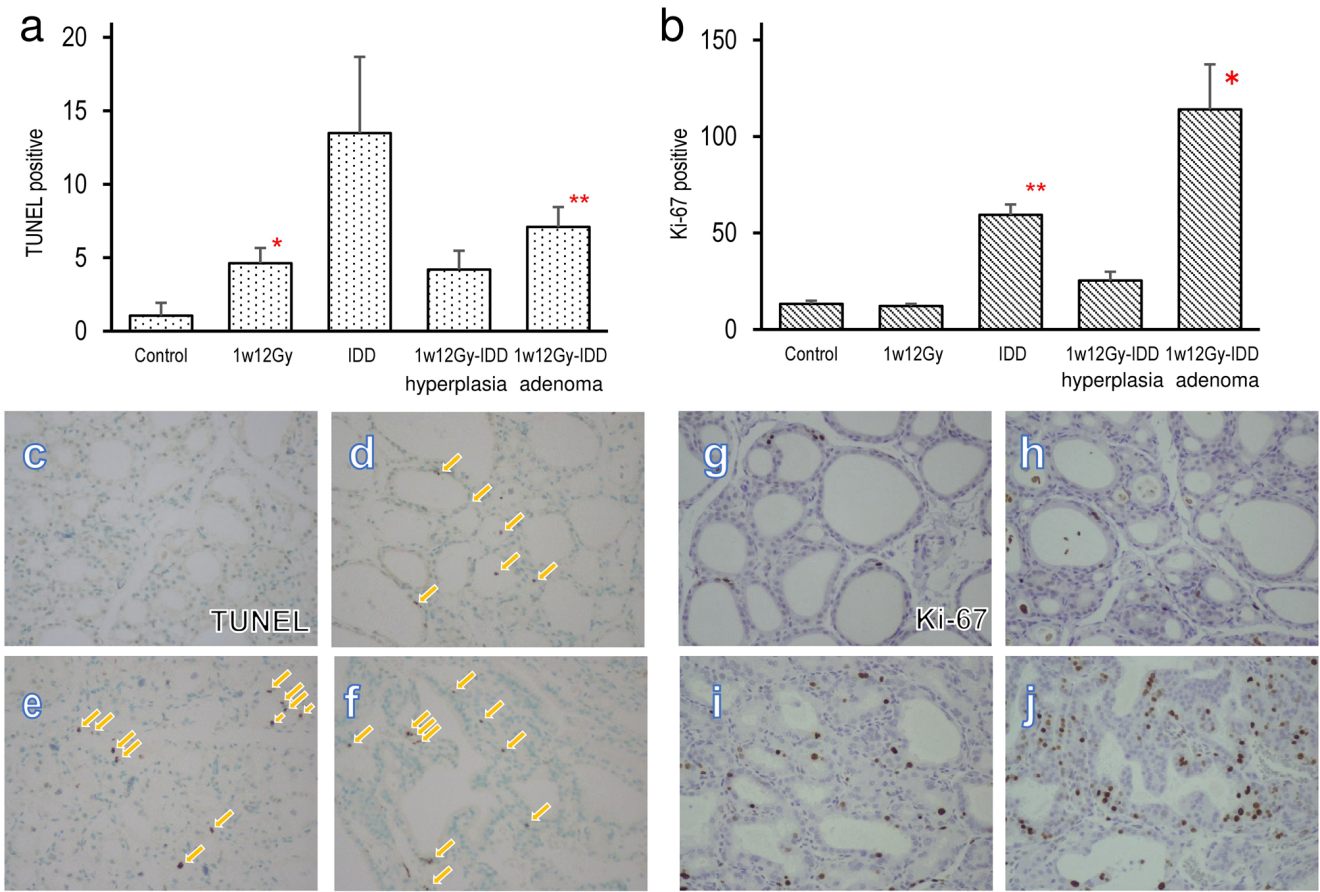
TUNEL and Ki-67 staining in the thyroid hyperplasia and adenoma of the neonatally X-irradiated rats fed with IDD. The numbers of TUNEL (**a**) or Ki-67 (**b**) positive cells in the thyroid of 29-week-old rats in Control, 1w12Gy, IDD, and 1w12Gy-IDD (hyperplasia and adenoma regions). Representative TUNEL (**c–f**) and Ki-67 (**g–j**) staining in Control (**c**, **g**), 1w12Gy (**d**, **h**), IDD (**e**, **i**), and 1w12Gy-IDD, adenoma region (**f**, **j**). Arrows indicate TUNEL positive staining. *, ** indicate significant difference from Control (*p < 0.05, **p < 0.01).

Figure 3 shows HE staining of the neonatally X-irradiated thyroid in rats fed with a standard diet or IDD. The size of thyroid follicles in the 1w12Gy group was still smaller than that in the Control at 29 weeks old (Fig. 3a and b). In the IDD group, thyroids of all animals became diffused follicular hyperplasia (Fig. 3c). In the 1w12Gy-IDD group, thyroid adenoma was observed at an incidence rate of 75% (n = 6/8), comprising one papillary and five follicular adenomas (Fig. 3d). The adenomatous regions were observed together with follicular hyperplasia in each histological section in the 1w12Gy-IDD group. Adenomas were diagnosed by expansive growth with basophilic cytoplasm and slightly atypical nuclear easily distinguished from the surrounding area, yet the lesions exhibited various patterns. The TUNEL and Ki-67 staining results are presented in Fig. 4. The numbers of TUNEL-positive cells were significantly increased in the 1w12Gy and 1w12Gy-IDD groups compared to the Control. Ki-67 positive cells were also higher in the IDD and 1w12Gy-IDD groups compared to Control. The adenomatous area was compared with the hyperplastic area in the 1w12Gy-IDD group, suggesting that both TUNEL and Ki-67 positive cells were increased in the adenomatous area.

### Expression of *Fas*, *Mki67*, *Mct8*, *Lat3*, *Met*, and *Lgals3* mRNAs in the X-irradiated thyroid

The mRNA levels of the examined genes of all experiments are summarized in Table 2. In Experiment 1, *Fas* mRNA levels increased dose-dependently post-irradiation, being significantly higher in 1w6Gy and 1w12Gy groups than the Control. *Lat4* mRNA levels decreased dose-dependently post-irradiation, while *Mct8* expression decreased only in the 1w12Gy. The *Lgals3* mRNA level was significantly elevated in the 1w12Gy group. In Experiment 2, Fas mRNA expression was significantly higher than Control but only in the neonatally irradiated group, 1w12Gy. The expression in *Lat4* and *Mct8* decreased in the 1w12Gy group. In experiment 3, *Fas* and *Lat4* expressions were still altered 28 weeks after X-irradiation (1w12Gy group). The IDD feeding led to substantial impacts on the expression of examined genes, suppressing *Mct8* and *Lat4* and increasing *Fas*, *Mki67*, *Met*, and *Lgals3*. The expression of *Lgals3* in 1w12Gy-IDD was higher than that in the IDD group.

**Table 2:** mRNA expression of *Fas*, *Mki67*, *Mct8*, *Lat4*, *Met*, and *Lgals3* in the thyroid of cervically X-irradiated rats.

Group	Apoptosis/proliferation		Differentiation markers		PTC markers	
	*Fas*	*Mki67(*Ki-67)	*Mct8*	*Lat4*	*Met*	*Lgals3*
**Experiment 1**						
Control	35 ± 4.0	62 ± 14.4	18 ± 2.1	14 ± 2.0	11 ± 0.9	40 ± 12.1
1w1.5 Gy	38 ± 1.8	48 ± 2.9	22 ± 1.7	11 ± 1.9	11 ± 0.7	27 ± 1.9
1w3Gy	60 ± 9.0	85 ± 14	17 ± 1.3	7.9 ± 1.3*	10 ± 0.6	56 ± 10.3
1w6Gy	52 ± 4.5*	60 ± 5.0	18 ± 2.1	7.8 ± 1.2*	11 ± 0.9	42 ± 5.4
1w12Gy	121 ± 9.8**	99 ± 17	12 ± 0.8*	3.9 ± 0.5**	11 ± 0.8	79 ± 13.3*
**Experiment 2**						
Control	28 ± 1.0	31 ± 2.1	17 ± 1.0	19 ± 3.6	10 ± 1.0	26 ± 2.3
1w12Gy	51 ± 2.0**	28 ± 1.9	13 ± 0.7*	6.1 ± 0.9*	8.5 ± 0.5	33 ± 3.1
2w12Gy	36 ± 4.7	27 ± 4.5	17 ± 1.5	10 ± 1.5	8.6 ± 0.4	24 ± 1.8
4w12Gy	63 ± 12	35 ± 3.1	11 ± 1.6*	9.3 ± 2.4	9.1 ± 0.8	42 ± 6.7
8w12Gy	29 ± 2.6	33 ± 3.4	18 ± 1.5	21 ± 3.6	8.7 ± 0.4	44 ± 6.6*
**Experiment 3**						
Control	4.6 ± 0.9	15 ± 1.3	29 ± 5.1	40 ± 6.9	5.8 ± 0.5	23 ± 2.8
1w12Gy	14 ± 1.2**	18 ± 0.3	38 ± 7.4	23 ± 3.5*	7.9 ± 0.7	52 ± 11.0
IDD	21 ± 2.5**	29 ± 2.7**	18 ± 2.6**	10 ± 1.9**	14 ± 1.1**	48 ± 5.1*
1w12Gy-IDD	21 ± 2.5**	28 ± 2.4**	19 ± 1.7**	8.7 ± 0.6**	17 ± 1.8**	64 ± 6.6**

*, and ** indicate significant differences from each control (*p < 0.05; **p < 0.01).

### Serum TT3, TT4, and TSH

Serum hormone levels are summarized in Table 3. In Experiment 1, serum TSH increased in the 1w12Gy groups while TT3 decreased in the 1w6Gy group. These changes were not observed at 17 weeks old in Experiment 2. Feeding IDD dramatically affected the hormonal status at 29 weeks old (Experiment 3). TT4 levels were dropped to one-tenth of the control level, and TSH increased by 18 times in the IDD and 1w12Gy-IDD groups. However, there were no differences among the TT3, TT4, or TSH levels of the IDD and the 1w12Gy-IDD groups.

**Table 3:** Serum TT3, TT4 and TSH levels in rats exposed to cervical X-irradiation.

Group	Total T3 (ng/ml)	Total T4 (µg/dl)	TSH (ng/ml)
**Experiment 1**			
Control	3.3 ± 0.23	5.4 ± 0.19	3.7 ± 0.31
1w1.5 Gy	2.9 ± 0.22	6.0 ± 0.41	3.7 ± 0.28
1w3Gy	2.9 ± 0.27	5.1 ± 0.46	4.0 ± 0.22
1w6Gy	2.5 ± 0.18*	5.1 ± 0.50	4.7 ± 0.29
1w12Gy	2.7 ± 0.17	4.7 ± 0.40	7.1 ± 0.66**
**Experiment 2**			
Control	2.4 ± 0.21	6.8 ± 0.45	5.6 ± 0.78
1w12Gy	2.2 ± 0.20	5.1 ± 0.69	6.7 ± 0.55
2w12Gy	2.0 ± 0.13	6.8 ± 0.75	7.0 ± 0.26
4w12Gy	2.0 ± 0.19	6.1 ± 0.74	6.9 ± 0.35
8w12Gy	1.7 ± 0.15	7.4 ± 0.48	7.5 ± 0.58
**Experiment 3**			
Control	2.3 ± 0.28	6.5 ± 0.72	4.7 ± 0.36
1w12Gy	2.2 ± 0.16	5.0 ± 0.68	5.1 ± 0.29
IDD	2.4 ± 0.18	0.5 ± 0.04**	71 ± 6.2**
1w12Gy-IDD	2.0 ± 0.18	0.7 ± 0.13**	82 ± 9.1**

*, and ** indicate significant differences from each control (*p < 0.05; **p < 0.01).

## Discussion

The present study demonstrated that single irradiation in the neonatal period led to the long-term changes in gene expressions and the dynamics of cell proliferation and death in the thyroid, which is probably responsible for the reduction in the size of the thyroid follicles. These changes may be related to thyroid tumorigenesis. It has been well established that radiation exposure at young ages is a risk of thyroid cancer^1–3,16,17^. Recently, a pooled analysis of 12 studies of thyroid cancer patients showed that those treated with radiation at ages of 0–1 year exhibited a 5.5-fold higher relative risk than those exposed at ages of 15–19 years^1,7^. The rat model of radiation-induced thyroid cancer was extensively studied in the 1960s and the 1970s^11,18–20^. A study on rats exposed to X-irradiation at the neck region demonstrated that neonatal rats (10 days old) exhibited higher susceptibility to the development of radiation-induced thyroid tumors than adult rats^13^. However, laboratory investigations with animal models have rarely been conducted to understand the underlying mechanisms. We recently demonstrated that the thyroid in rats X-irradiated at the age of 5–6 days developed the specific morphological changes characterized by smaller-sized follicles^15^. In the present study, we further investigated this model to understand the impacts of neonatal irradiation on the thyroid.

We first examined the dose- and age-dependency of the effects of X-irradiation. The thyroid follicular size was reduced following neonatal irradiation in a dose-dependent manner. The data also showed that the effect was only evident with the irradiation during the neonatal period, as there were no signs of changes in thyroid morphology in rats X-irradiated at the age of 2 weeks or older. In rats, the mitotic activity of the thyroid follicular cells becomes the maximum during the postnatal days 5–10^21^. The sensitivity to X-irradiation may decline after this point. Further studies are required to understand when the critical neonatal period exists for X-irradiation inducing thyroid abnormalities. Despite these morphological changes, serum TT3 and TSH levels only altered at the age of 9 weeks, which was consistent with our previous study that showed a similar temporal increase in the serum TSH level^15^. TUNEL staining indicated that the number of apoptotic cells increased in the neonatally irradiated thyroid, while Ki-67 staining showed the cell proliferation was not affected by irradiation. From the neonate to the adult stage, thyroid follicular size increases continuously as the thyroid gland matures^21^. Because the neonatal irradiation induced continuous cell death in the follicular epithelium, the normal growth of thyroid follicles was probably interrupted, maintaining the follicular sizes small. FAS is a membrane-associated protein and is involved in the apoptotic pathway^22^. A rat model of goiter showed that the elevation of FAS protein expression was associated with an increased number of apoptotic cells^23^. In the present study, a single X-irradiation at the neonatal period apparently induced the long-term elevation in *Fas* gene expression resulting in the continuous apoptotic cell death in the thyroid. The Ki-67 staining and its mRNA expression levels indicated no significant changes in proliferative activities by the neonatal X-irradiation.

To examine whether the single neonatal X-irradiation alters the expressions of other genes, we measured some of the thyroid cancer-related expression markers, including *Mct8*, *Lat4*, *Met*, and *Lgals3*. MCT8 and LAT4 are thyroid hormone transporters localized in the plasma membrane of thyroid epithelial cells^24^. An immunohistochemical study in human specimens showed that these transporters' expressions were downregulated in thyroid adenoma and carcinoma, reflecting their malignancy^25^. The neonatal X-irradiation decreased the expressions of these genes. *Lat4* appeared to be more radiation-sensitive as its expression reduced at 3 Gy. The reduced expression of these markers may indicate that the thyroid remained undifferentiated and may be susceptible to tumor development. There have been many microarray studies to identify the differentially expressed genes in human thyroid cancer^10,26^. A recent study combining all these data identified the thyroid cancer-specific upregulated genes, such as *Met*, and *Lgals3*, which indicated common oncogenic pathways for this cancer^10^. MET is a tyrosine kinase receptor involving invasive growth functioning both in the physiological process during embryogenesis and pathological actions during cancer progression^27^. LGALS3 or galectin-3 is a member of the chimeric galectin subfamily capable of interacting with galactose-containing oligosaccharides^28^. A study of thyroid cell culture suggested a pivotal role of *Lgals3* gene in the transformation of thyroid follicular cells^29^. The continuous upregulation of *Lgals3* gene expression by neonatal irradiation may also be related to thyroid tumorigenesis.

In rodent models, the development of thyroid tumors is strongly associated with an increased level of serum TSH^30^. Chemical carcinogenesis studies in rats fed with IDD showed that TSH played a role mainly as a promoter in thyroid cancer development^31–33^. To analyze the possible relationship between neonatal X-irradiation and thyroid tumor development, we examined the thyroid tumor model in rats fed with IDD. The thyroid adenoma developed at an incidence rate of 75% (n = 6/8) in the neonatal X-irradiated IDD group, while hyperplasia was solely observed without irradiation. Our results were consistent with an early study on X-irradiated neonatal rats treated with an antithyroid chemical, 4-methyl-2-thiouracil (MTU), where the incidence rate of thyroid adenoma reached 80% in 7 months after the exposure, although carcinoma started to emerge in 9 months^13^. Similar to our results, the induced tumors were predominantly of a follicular type. Interestingly, the IDD feeding alone altered the expressions of all the genes examined, indicating its tumor promoter activity. Nevertheless, the IDD feeding evidently suppressed the thyroid hormone production and increased serum TSH levels, which, in turn, stimulated the proliferation of thyroid follicular cells. The TUNEL staining results indicated that neonatal X-irradiation continuously increased the number of apoptotic cells in the thyroid even at 28 weeks post-exposure. The combination of the elevated apoptotic activity by irradiation and the increased proliferation by IDD seems to contribute to the tumorigenesis. The gene expression data showed that IDD further enhanced the upregulation of *Lgals3* caused by neonatal irradiation, which may also be related to the development of thyroid adenoma.

## Conclusions

Single neonatal irradiation induced continuous changes in expressions of the thyroidal gene, which was similar to the tumor promotion effects induced by IDD. There long-term changes in gene expression may be the key to explaining the underlying mechanisms of thyroid carcinogenesis due to childhood radiation. Further investigations are still warranted to understand the molecular mechanisms of related gene regulation.

## Methods

### Animal studies

**Figure 5:**
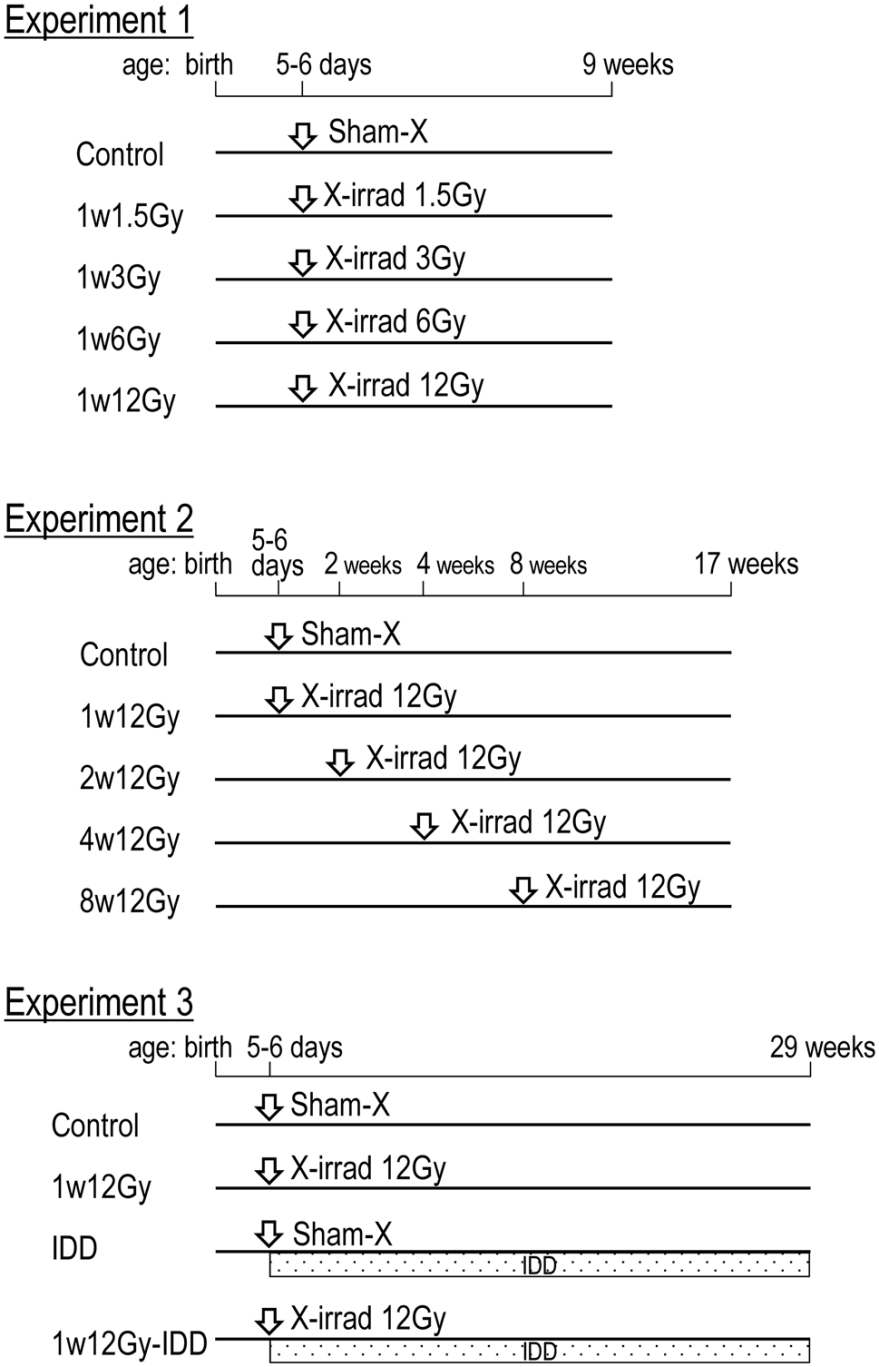
Schedule of the experiments. In Experiment 1 (a radiation dose–response study), rats were exposed to sham, 1.5, 3, 6, or 12 Gy of cervical X-irradiation at 5 or 6 days old and necropsied at 9 weeks old. In experiment 2 (a study of irradiation at different ages), 12 Gy of cervical X-irradiation was given at 5 or 6 days, 2, 4, or 8 weeks old. In Experiment 3 (a thyroid tumor promotion study), rats were exposed to 12 Gy of cervical X-irradiation at 5 or 6 days old. After the exposure, half of the groups were fed with IDD (The mothers were fed with IDD until weaning at 3.5 weeks old).

Three-month-old pregnant Wistar rats (16–18 days of gestation) were purchased from Charles River Laboratories Japan, Inc. (Kanagawa, Japan). Rats were maintained with free access to a basal diet (MF, Oriental Yeast Co., Tokyo, Japan) and tap water. The animal facility conditions were as follows: room temperature of 23.0 ± 2.0 °C, a relative humidity of 50.0 ± 10.0%, and a 12-h light cycle. After delivery, male pups were selected for the study. The schedule of animal experiments is summarized in Fig. 5. In Experiment 1 (a radiation dose–response study), the pups were randomly divided into five groups (6 rats per group). The groups were then exposed to sham, 1.5, 3, 6, or 12 Gy of cervical X-irradiation at 5 or 6 days old and designated as Control, 1w1.5 Gy, 1w3Gy, 1w6Gy, and 1w12Gy, respectively. Animals were killed by whole blood removal from an abdominal artery under anesthesia induced by isoflurane at 9 weeks old. In experiment 2 (a study of irradiation at different ages), pops were randomly divided into 5 groups (5 rats per group). The groups were then exposed to 12 Gy of cervical X-irradiation at 5 or 6 days, and 2, 4, and 8 weeks old, while one group was sham-irradiated at 5–6 days old and designated as 1w12Gy, 2w12Gy, 4w12Gy, 8w12Gy, and Control, respectively. All the animals were killed at the age of 17 weeks old. In Experiment 3 (a thyroid tumor promotion study), pups were randomly divided into four groups and exposed to sham or 12 Gy of cervical X-irradiation at 5 or 6 days old. After the exposure, half of the groups were fed with IDD. The groups were then designated as Control, 1w12Gy (X-irradiation only), IDD (IDD only), and 1w12Gy-IDD (X-irradiation plus IDD). Animals were killed at the age of 29 weeks. For each experiment, the left lobes of the thyroid tissues were stored in RNA Save solution (Biological Industries, Cromwell, CT, USA) for RNA extraction, and the other lobes were fixed in 10% formalin for histological examination. The animal experiment was approved by the Animal Experiment Committee of Hiroshima University (document # A19-69) and was conducted in accordance with the Guide for the Care and Use of Laboratory Animals at Hiroshima University. The study was carried out in compliance with the ARRIVE guidelines.

### X-irradiation

An X-ray irradiator, MBR-1520R-3 (Hitachi Medical Co., Tokyo, Japan), was used. The dose rate was approximately 0.9 Gy/min. During irradiation, rats were under anesthesia induced by intraperitoneal injection of an anesthetic mixture: 0.3 mg/kg of medetomidine, 4.0 mg/kg of midazolam, and 5.0 mg/kg of butorphanol; and placed with the ventral side up from which X-irradiation was given. Two plates of lead of 2 mm thickness were used to cover each animal, leaving a gap for the neck region. For sham irradiation, rats were entirely covered with lead plates and irradiated.

### Histological analyses

Thyroid tissues were fixed in 10% formalin and embedded in paraffin. The 4-μm-thick sections were stained with hematoxylin and eosin (HE). The colloid areas were measured using the particle analysis function of ImageJ software (http://imagej.nih.gov). TUNEL staining was performed with the ApopTag Peroxidase In Situ Apoptosis Detection Kit (Merck KGaA, Darmstadt, Germany), following the manufacturer's protocol. For immunohistochemical staining for Ki-67, a polyclonal rabbit antibody to Ki-67 (ab15580, at 1:5,000 delusion) and Simple Stain Rat Max-PO (Nichirei Bioscience, Tokyo, Japan) were used. The TUNEL-positive and Ki-67-positive cells were counted in five fields per rat.

### Quantification of mRNA levels by quantitative RT-PCR

Total RNA was prepared using Isogen II (Nippon Gene Co., Tokyo, Japan) from the thyroid tissue stored in RNA Save solution. The cDNA was synthesized by incubating 3 µg total RNA with 100 U of ReverTra Ace reverse transcriptase (Toyobo Co., Osaka, Japan) with a mixture of 20 pmol random hexamers pdN6 and 5 pmol oligo-dT(15) primers (Takara Bio Inc., Kusatsu, Japan). A quantitative PCR instrument, StepOnePlus (Applied Biosystems/Life Technologies Co., Carlsbad, CA, USA), was used to measure cDNA levels with a KAPA SYBR Fast qPCR Kit (Kapa Biosystems, Inc., Woburn, MA, USA). The sequences of specific primer sets are listed in Table 4. The DNA sequences of the PCR products were confirmed by Fasmac Co., Ltd. (Atsugi, Japan). The PCR conditions were as follows: 30 s initial denaturation, followed by 40 cycles of 5 s-incubation at 95 °C and 35 s-incubation at 60 °C. The measured mRNA levels were normalized against the levels of β-actin mRNA^34^.

**Table 4:** Q-PCR primers.

Gene	GenBank Accession#	Q-PCR primer sequences (5'—> 3')	
		Forward	Reverse
*Fas*	NM_139194	AAGATCGATGAGATCGAGCACA	GCAGCGGTTAGCTTTTCTGAGA
*Mki67*	NM_001271366	GCAACCTTTACCTGTCCCCC	TGGTAGAAGCTGCCCTTTGTTT
*Mct8*	NM_147216	GTTTGAACTGGTGGGACCCAT	GACACCCGCAAAGTAGAAGGC
*Lat4*	NM_053442	CACATTTGGTGGAGTCAACGG	GGTGGAGAGGCATGTGAAGAG
*Met*	NM_031517	AGACGCAAAAGTTCACCACCA	GGTTGCAAGAGTCTTCTGCCTT
*Lgals3*	NM_031832	CCCGCTTCAATGAGAACAACA	AACCTTGAAGTGGTCGGCTTC

### Statistical analysis

All values are expressed as mean ± standard error (S.E.). Dunnett's test was applied for multiple comparisons among groups.

## Supplementary Information

Supplementary Information.
