# Supplementary material for: Single neonatal irradiation induces long-term gene expression changes in the thyroid gland, which may be involved in the tumorigenesis
Source: Sci Rep. 2021 Dec 8;11:23620. doi: 10.1038/s41598-021-03012-5 (PMC8655036; doi:10.1038/s41598-021-03012-5)
Supplement: Supplementary file 1 — Supplementary Information. [file 41598_2021_3012_MOESM1_ESM.pdf]

Supplemental Information

Single neonatal irradiation induces long-term gene expression changes and cell death in the thyroid gland, which may be involved in the tumorigenesis

Nariaki Fujimoto,[1,*] Mutsumi Matsuu-Matsuyama[2] & Masahiro Nakashima[2,3]

[1]Institute for Radiation Biology and Medicine (RIRBM), Hiroshima University, Hiroshima, Japan

[2]Tissue and Histopathology Section, Atomic Bomb Disease Institute, Nagasaki University, Nagasaki, Japan

[3]Department of Tumor and Diagnostic Pathology, Atomic Bomb Disease Institute, Nagasaki University, Nagasaki, Japan

** nfjm@hiroshima-u.ac.jp*

ARRIVE

The ARRIVE Guidelines Checklist

Animal Research: Reporting In Vivo Experiments

Carol Kilkenny[1], William J Browne[2], Innes C Cuthill[3], Michael Emerson[4] and Douglas G Altman[5]

[1]The National Centre for the Replacement, Refinement and Reduction of Animals in Research, London, UK, [2]School of Veterinary Science, University of Bristol, Bristol, UK, [3]School of Biological Sciences, University of Bristol, Bristol, UK, [4]National Heart and Lung Institute, Imperial College London, UK, [5]Centre for Statistics in Medicine, University of Oxford, Oxford, UK.

	ITEM	RECOMMENDATION	Section/ Paragraph
Title	1	Provide as accurate and concise a description of the content of the article as possible.	Title
Abstract	2	Provide an accurate summary of the background, research objectives, including details of the species or strain of animal used, key methods, principal findings and conclusions of the study.	Abstract
INTRODUCTION			
Background	3	a. Include sufficient scientific background (including relevant references to previous work) to understand the motivation and context for the study, and explain the experimental approach and rationale.<br>b. Explain how and why the animal species and model being used can address the scientific objectives and, where appropriate, the study's relevance to human biology.	P4L2-12<br>P4L16-P5L1
Objectives	4	Clearly describe the primary and any secondary objectives of the study, or specific hypotheses being tested.	P5L1-6
METHODS			
Ethical statement	5	Indicate the nature of the ethical review permissions, relevant licences (e.g. Animal [Scientific Procedures] Act 1986), and national or institutional guidelines for the care and use of animals, that cover the research.	P13L13-15
Study design	6	For each experiment, give brief details of the study design including:<br>a. The number of experimental and control groups.<br>b. Any steps taken to minimise the effects of subjective bias when allocating animals to treatment (e.g. randomisation procedure) and when assessing results (e.g. if done, describe who was blinded and when).<br>c. The experimental unit (e.g. a single animal, group or cage of animals).<br>A time-line diagram or flow chart can be useful to illustrate how complex study designs were carried out.	P12L17-P13L9<br>P13L1-3<br>Table 1
Experimental procedures	7	For each experiment and each experimental group, including controls, provide precise details of all procedures carried out. For example:<br>a. How (e.g. drug formulation and dose, site and route of administration, anaesthesia and analgesia used [including monitoring], surgical procedure, method of euthanasia). Provide details of any specialist equipment used, including supplier(s).<br>b. When (e.g. time of day).<br>c. Where (e.g. home cage, laboratory, water maze).<br>d. Why (e.g. rationale for choice of specific anaesthetic, route of administration, drug dose used).	P12L13-17<br>Table1
Experimental animals	8	a. Provide details of the animals used, including species, strain, sex, developmental stage (e.g. mean or median age plus age range) and weight (e.g. mean or median weight plus weight range).<br>b. Provide further relevant information such as the source of animals, international strain nomenclature, genetic modification status (e.g. knock-out or transgenic), genotype, health/immune status, drug or test naïve, previous procedures, etc.	P12L13-14

Housing and husbandry	9	Provide details of:<br>a. Housing (type of facility e.g. specific pathogen free [SPF]; type of cage or housing; bedding material; number of cage companions; tank shape and material etc. for fish).<br>b. Husbandry conditions (e.g. breeding programme, light/dark cycle, temperature, quality of water etc for fish, type of food, access to food and water, environmental enrichment).<br>c. Welfare-related assessments and interventions that were carried out prior to, during, or after the experiment.	P12L13-17
Sample size	10	a. Specify the total number of animals used in each experiment, and the number of animals in each experimental group.<br>b. Explain how the number of animals was arrived at. Provide details of any sample size calculation used.<br>c. Indicate the number of independent replications of each experiment, if relevant.	P12L17-P13L9
Allocating animals to experimental groups	11	a. Give full details of how animals were allocated to experimental groups, including randomisation or matching if done.<br>b. Describe the order in which the animals in the different experimental groups were treated and assessed.	P12L17-P13L8
Experimental outcomes	12	Clearly define the primary and secondary experimental outcomes assessed (e.g. cell death, molecular markers, behavioural changes).	P8L14-16
Statistical methods	13	a. Provide details of the statistical methods used for each analysis.<br>b. Specify the unit of analysis for each dataset (e.g. single animal, group of animals, single neuron).<br>c. Describe any methods used to assess whether the data met the assumptions of the statistical approach.	P15L8-9
**RESULTS**			
Baseline data	14	For each experimental group, report relevant characteristics and health status of animals (e.g. weight, microbiological status, and drug or test naïve) prior to treatment or testing. (This information can often be tabulated).	Suppl_Data & Table 1
Numbers analysed	15	a. Report the number of animals in each group included in each analysis. Report absolute numbers (e.g. 10/20, not 50%[2]).<br>b. If any animals or data were not included in the analysis, explain why.	Suppl_Data & Table 1
Outcomes and estimation	16	Report the results for each analysis carried out, with a measure of precision (e.g. standard error or confidence interval).	Tables1-3/Figures
Adverse events	17	a. Give details of all important adverse events in each experimental group.<br>b. Describe any modifications to the experimental protocols made to reduce adverse events.	No adverse events
**DISCUSSION**			
Interpretation/ scientific implications	18	a. Interpret the results, taking into account the study objectives and hypotheses, current theory and other relevant studies in the literature.<br>b. Comment on the study limitations including any potential sources of bias, any limitations of the animal model, and the imprecision associated with the results[2].<br>c. Describe any implications of your experimental methods or findings for the replacement, refinement or reduction (the 3Rs) of the use of animals in research.	P11L16-P12L3<br>P12L6-10
Generalisability/ translation	19	Comment on whether, and how, the findings of this study are likely to translate to other species or systems, including any relevance to human biology.	P10L16-P11L4, P12L6-9
Funding	20	List all funding sources (including grant number) and the role of the funder(s) in the study.	'Acknowledgments'

References:

1. Kilkenny C, Browne WJ, Cuthill IC, Emerson M, Altman DG (2010) Improving Bioscience Research Reporting: The ARRIVE Guidelines for Reporting Animal Research. *PLoS Biol* 8(6): e1000412. doi:10.1371/journal.pbio.1000412
2. Schulz KF, Altman DG, Moher D, the CONSORT Group (2010) CONSORT 2010 Statement: updated guidelines for reporting parallel group randomised trials. *BMJ* 340:c332.

Table 1 Data

Exp1		BW			Thyroid			Thyroid/bw		
Control	W221	398			24			60		
	W222	369			22			60		
	W223	401			16			40		
	W224	381			20			52		
	W225	343	Mean	SE	16	Mean	SE	47	Mean	SE
	W226	390	380	8.9	12	18	1.8	31	48	4.7
1wX-1.5Gy	W227	424			22			52		
	W228	394			24			61		
	W229	399			16			40		
	W230	348			18			52		
	W231	382			16			42		
	W232	395	390	10.2	18	19	1.3	46	49	3.2
1wX-3Gy	W233	384			16			42		
	W234	334			22			66		
	W235	334			18			54		
	W236	364			12			33		
	W237	351			14			40		
	W238	360	355	7.8	20	17	1.5	56	48	5.0
1wX-6Gy	W239	373			16			43		
	W240	282			10			35		
	W241	319			14			44		
	W242	290			12			41		
	W243	309			12			39		
	W244	405	330	19.9	20	14	1.5	49	42	1.9
1wX-12Gy	W245	220			10			45		
	W246	242			10			41		
	W247	272			16			59		
	W248	345			12			35		
	W249	361			14			39		
	W250	304	291	22.9	12	12	1.0	39	43	3.4
Exp2										
Control	W251	623			36			58		
	W252	659			28			42		
	W253	540			30			56		
	W254	604			28			46		
	W255	610	607	19.3	22	29	2.2	36	48	4.0
1wX-12Gy	W256	409			8			20		
	W257	512			18			35		
	W258	560			14			25		
	W259	370			16			43		
	W260	378	446	38.2	18	15	1.9	48	34	5.3
2wX-12Gy	W261	516			20			39		
	W262	518			20			39		
	W263	589			26			44		
	W264	606	557	23.5	28	24	2.1	46	42	1.9
4wX-12Gy	W265	501			22			44		
	W266	568			20			35		
	W267	600			20			33		
	W268	499			20			40		
	W269	710	576	38.8	22	21	0.5	31	37	2.3
8wX-12Gy	W270	537			22			41		
	W271	559			28			50		
	W272	562			20			36		
	W273	501			24			48		
	W274	547	541	11.0	28	24	1.6	51	45	3.0
Exp3	TT3									
Control	W191	721			16			22		
	W192	698			32			46		
	W193	681			16			23		
	W194	668	692	11.5	18	21	3.9	27	30	5.5
1wX-12Gy	W195	592			16			27		
	W196	529			12			23		
	W197	445	522	42.6	16	15	1.3	36	29	3.9
IDD	W198	275			284			1033		
	W199	301			210			698		
	W200	320			198			619		
	W201	278			416			1496		
	W202	290			350			1207		
	W203	304			268			882		
	W204	320	298	6.9	326	293	29.3	1019	993	113.5
1wX-12Gy-IDI	W205	213			140			657		
	W206	218			102			468		
	W207	271			164			605		
	W208	316			280			886		
	W209	182			136.8			752		
	W210	248			128			516		
	W211	253			146			577		
	W212	292	249	15.6	80	147	21.1	274	592	65.3

Dunnet test	BW	Thyroid	Thyroid/bw
Exp1	p.value.adj	p.value.adj	p.value.adj
1wX-1.5Gy vs C	0.978	0.837	0.776
1wX-3Gy vs C	0.092	0.524	0.755
1wX-6Gy vs C	0.093	0.116	0.260
1wX-12Gy vs C	0.018	0.029	0.377
Exp2			
1wX-12Gy	0.039	0.006	0.204
2wX-12Gy	0.396	0.291	0.538
4wX-12Gy	0.888	0.059	0.147
8wX-12Gy	0.076	0.336	0.949
Exp3			
1wX	0.121	0.498	0.999
IDD	0.000	0.000	0.000
1wX-IDD	0.000	0.002	0.000

Table 2 Data - QPCR

		bactin	FAS	x1000/bact		KI67	x1000/bact		MCT8	x100/bact		LAT4		x100/bact	MET		x1000/bact	LGALS3	x1000/bact	
1wX-0Gy	W221	42.17	1.88	44.6		5.45	129.2		6.14	14.6		7.67	18.20		0.54	12.80		4.06	96.3	
	W222	79.19	2.61	33.0		3.34	42.1		20.50	25.9		14.19	17.92		0.88	11.14		2.23	28.2	
	W223	79.15	2.35	29.7		2.99	37.7		14.34	18.1		4.33	5.47		0.71	8.99		2.71	34.2	
	W224	95.34	2.09	21.9		3.38	35.5		14.17	14.9		10.88	11.41		0.70	7.39		2.16	22.7	
	W225	45.71	2.20	48.1	Mean	2.85	62.3		10.71	23.4		7.94	17.37		0.58	12.65		1.84	40.2	
	W226	70.90	2.32	32.7	35.0	4.46	62.8	61.6	9.71	13.7	18.4	9.05	12.77	13.9	0.75	10.56	10.6	1.33	18.8	40.1
					3.97			14.37			2.09			2.04			0.86			11.69
1wX-1.5Gy	W227	66.39	2.62	39.5	SE	3.33	50.1		13.97	21.0		9.42	14.18		0.88	13.25		1.65	24.8	
	W228	57.34	2.44	42.6		3.08	53.7		14.18	24.7		3.41	5.94		0.59	10.33		1.95	33.9	
	W229	63.73	2.09	32.8		3.62	56.9		17.46	27.4		10.31	16.18		0.83	13.02		1.41	22.1	
	W230	67.85	2.74	40.3		2.69	39.7		12.77	18.8		7.52	11.08		0.74	10.87		1.98	29.2	
	W231	76.44	3.10	40.5		3.54	46.3		18.26	23.9		10.59	13.86		0.79	10.36		1.88	24.6	
	W232	72.64	2.36	32.5	38.0	2.92	40.3	47.8	11.37	15.6	21.9	3.79	5.22	11.1	0.61	8.42	11.0	1.81	25.0	26.6
					1.76			2.87			1.75			1.86			0.74			1.74
1wX-3Gy	W233	42.44	2.76	65.0		2.54	59.8		5.18	12.2		5.20	12.26		0.44	10.43		3.10	73.1	
	W234	61.73	2.97	48.1		3.67	59.4		13.24	21.4		3.31	5.37		0.55	8.95		2.27	36.7	
	W235	36.23	2.43	67.2		3.05	84.3		6.24	17.2		4.06	11.20		0.43	11.83		3.10	85.5	
	W236	86.91	3.04	34.9		4.75	54.7		15.42	17.7		5.20	5.98		0.71	8.22		3.16	36.4	
	W237	76.11	3.73	49.0		9.70	127.4		12.14	15.9		5.92	7.78		0.77	10.13		2.17	28.5	
	W238	27.99	2.75	98.3	60.4	3.53	126.0	85.3	5.43	19.4	17.3	1.31	4.70	7.9	0.32	11.33	10.1	2.18	78.0	56.4
					9.01			13.77			1.28			1.30			0.56			10.26
1wX-6Gy	W239	47.10	3.35	71.2		3.48	73.8		5.02	10.7		4.50	9.56		0.45	9.65		3.01	63.9	
	W240	57.99	2.82	48.7		4.04	69.7		9.35	16.1		5.74	9.90		0.63	10.94		1.82	31.4	
	W241	64.98	3.70	57.0		3.67	56.4		16.37	25.2		6.17	9.50		0.83	12.76		2.70	41.6	
	W242	58.85	2.34	39.7		2.49	42.4		9.18	15.6		2.28	3.87		0.47	7.94		2.52	42.9	
	W243	61.14	3.12	51.0		3.08	50.3		13.71	22.4		2.37	3.87		0.58	9.56		2.73	44.6	
	W244	60.76	2.71	44.7	52.0	4.11	67.6	60.0	11.78	19.4	18.2	6.02	9.90	7.8	0.86	14.15	10.8	1.56	25.7	41.7
					4.52			5.03			2.13			1.23			0.93			5.36
1wX-12Gy	W245	61.91	6.44	104.1		4.51	72.9		9.89	16.0		3.59	5.80		0.82	13.20		2.51	40.6	
	W246	71.14	6.58	92.4		3.31	46.5		9.19	12.9		1.72	2.41		0.88	12.39		4.13	58.0	
	W247	30.41	4.75	156.1		3.65	120.1		3.21	10.6		1.30	4.29		0.31	10.21		3.72	122.2	
	W248	43.21	5.55	128.3		4.62	106.9		4.94	11.4		1.41	3.26		0.34	7.81		3.23	74.7	
	W249	33.31	4.66	139.8		5.43	163.1		4.24	12.7		1.22	3.67		0.43	12.80		3.82	114.6	
	W250	39.94	4.32	108.2	121.5	3.46	86.7	99.4	4.54	11.4	12.5	1.68	4.20	3.9	0.49	12.19	11.4	2.59	64.7	79.1
					9.84			16.54			0.79			0.47			0.84			13.25

		bactin	FAS			KI67			MCT8			LAT4			MET			LGALS3		
C	W251	59.58	1.64	27.5		1.56	26.1		10.56	17.7		4.47	7.51		0.61	10.20		1.27	21.3	
	W252	66.33	2.00	30.1		2.43	36.7		11.13	16.8		14.64	22.07		0.81	12.23		2.20	33.1	
	W253	65.66	2.03	31.0		2.35	35.8		11.76	17.9		17.42	26.53		0.72	11.00		1.93	29.4	
	W254	80.93	2.10	25.9		2.23	27.6		14.84	18.3		19.83	24.50		0.59	7.33		1.78	22.0	
	W255	70.81	1.88	26.5	28.2	2.21	31.2	31.5	9.10	12.9	16.7	9.33	13.18	18.8	0.54	7.66	9.7	1.69	23.8	25.9
					1.00			2.12			1.00			3.62			0.95			2.29
1wX-12Gy	W256	95.16	4.68	49.2		2.20	23.2		14.37	15.1		6.63	6.97		0.67	7.04		2.27	23.9	
	W257	87.90	5.08	57.8		2.66	30.2		9.92	11.3		8.12	9.23		0.85	9.65		2.53	28.7	
	W258	80.28	4.37	54.4		1.91	23.8		11.13	13.9		3.60	4.48		0.63	7.82		2.89	36.0	
	W259	90.43	4.32	47.8		2.67	29.6		10.97	12.1		4.70	5.20		0.79	8.70		3.53	39.0	
	W260	97.13	4.62	47.5	51.3	3.20	32.9	27.9	13.85	14.3	13.3	4.55	4.68	6.1	0.90	9.22	8.5	3.86	39.8	33.5
					2.03			1.91			0.70			0.89			0.47			3.09
2wX-12Gy	W261	71.90	2.60	36.2		1.46	20.3		12.77	17.8		6.91	9.61		0.60	8.35		2.03	28.2	
	W262	80.69	3.77	46.7		1.69	20.9		15.01	18.6		10.41	12.90		0.62	7.71		1.93	23.9	
	W263	109.71	4.21	38.3		2.97	27.1		13.18	12.0		6.20	5.65		0.99	9.00		2.15	19.6	
	W264	77.56	1.85	23.8	36.3	3.08	39.8	27.0	13.95	18.0	16.6	8.65	11.15	9.8	0.72	9.29	8.6	1.96	25.2	24.2
					4.73			4.51			1.54			1.55			0.35			1.78
4wX-12Gy	W265	79.28	1.49	18.8		1.99	25.0		10.12	12.8		14.82	18.69		0.63	7.94		1.39	17.5	
	W266	99.69	6.44	64.6		4.19	42.0		10.23	10.3		6.34	6.36		0.67	6.75		4.19	42.1	
	W267	59.74	4.27	71.5		2.46	41.3		6.22	10.4		3.57	5.98		0.53	8.85		3.18	53.2	
	W268	55.75	4.94	88.5		1.80	32.3		4.02	7.2		4.06	7.29		0.63	11.36		3.06	54.9	
	W269	80.73	5.69	70.5	62.8	2.75	34.1	34.9	13.42	16.6	11.5	6.72	8.32	9.3	0.85	10.57	9.1	3.52	43.6	42.3
					11.70			3.13			1.56			2.37			0.84			6.68
8wX-12Gy	W270	73.37	2.33	31.7		1.72	23.4		13.08	17.8		10.17	13.86		0.66	9.00		3.34	45.5	
	W271	72.22	2.57	35.7		2.39	33.1		15.21	21.1		10.41	14.42		0.59	8.20		3.03	42.0	
	W272	63.74	1.50	23.6		1.85	29.0		8.05	12.6		12.10	18.99		0.48	7.56		1.56	24.5	
	W273	56.12	1.51	27.0		2.05	36.6		10.43	18.6		18.96	33.78		0.50	8.88		2.35	41.8	
	W274	77.45			29.5	3.35	43.3	33.1	15.49	20.0	18.0	17.57	22.69	20.7	0.78	10.04	8.7	5.11	65.9	44.0
					2.65			3.36			1.46			3.64			0.42			6.60

		bactin	FAS			KI67			MCT8			LAT4			MET			LGALS3		
C (29wo)	W191	50.24	0.33	6.6		0.63	12.5		12.94	25.8		15.56	30.97		0.30	6.02		1.51	30.1	
	W192	64.47	0.15	2.3		0.95	14.8		19.09	29.6		22.37	34.69		0.39	6.01		1.55	24.0	
	W193	66.78	0.37	5.6		1.22	18.2		28.17	42.2		35.60	53.31		0.46	6.85		1.19	17.9	
	W194	79.51	0.32	4.1	4.6	1.06	13.3	14.7	14.21	17.9	28.9			39.7	0.35	4.35	5.8	1.50	18.9	22.7
					0.94			1.28			5.07			6.91			0.52			2.80
1wX(29wo)	W195	56.65	0.77	13.7		1.05	18.6		18.21	32.1		16.35	28.87		0.49	8.65		2.17	38.3	
	W196	61.47	1.01	16.5		1.08	17.6		32.04	52.1		14.47	23.53		0.53	8.68		2.69	43.8	
	W197	23.69	0.29	12.5	14.2	0.42	17.6	17.9	6.72	28.4	37.5	3.92	16.53	23.0	0.15	6.45	7.9	1.72	72.7	51.6
					1.19			0.32			7.37			3.57			0.74			10.66
IDD (27wo)	W198	122.67	2.82	23.0		4.79	39.0		24.19	19.7		26.24	21.39		1.42	11.58		4.82	39.3	
	W199	110.48	2.75	24.9		4.31	39.0		24.64	22.3		11.11	10.05		1.50	13.55		6.16	55.8	
	W200	95.50	0.78	8.2		2.60	27.2		12.54	13.1		5.79	6.06		1.03	10.77		5.34	55.9	
	W201	101.66	1.72	17.0		2.65	26.1		21.38	21.0		10.87	10.69		1.94	19.12		4.09	40.3	
	W202	93.28	2.38	25.5		2.55	27.3		26.15	28.0		9.31	9.98		1.39	14.90		3.50	37.6	
	W203	112.84	3.05	27.1		2.34	20.7		16.01	14.2		8.18	7.25		1.37	12.13		4.08	36.1	
	W204	110.42	2.18	19.8	20.8	2.67	24.2	29.1	8.08	7.3	18.0	8.34	7.56	10.4	1.70	15.42	13.9	7.97	72.1	48.2
					2.48			2.70			2.60			1.94			1.08			5.10
1wX+IDD (27wo)	W205	103.97	3.16	30.3		3.01	29.0		24.16	23.2		10.53	10.13		1.71	16.45		6.32	60.8	
	W206	82.39	0.87	10.5		1.58	19.2		11.63	14.1		5.12	6.21		1.02	12.32		6.45	78.3	
	W207	87.33	1.28	14.6		2.00	22.9		10.61	12.1		6.80	7.79		1.21	13.91		7.32	83.9	
	W208	95.91	2.29	23.9		2.30	24.0		17.49	18.2		8.82	9.19		1.16	12.09		4.96	51.8	
	W209	132.44	2.17	16.4		4.65	35.1		33.17	25.0		11.99	9.06		1.55	11.72		9.75	73.6	
	W210	88.50	2.61	29.5		3.30	37.3		14.59	16.5		8.26	9.33		1.71	19.29		7.49	84.6	
	W211	83.07	1.82	22.0		2.85	34.3		20.13	24.2		9.12	10.98		2.10	25.22		3.65	44.0	
	W212	107.25	1.99	18.6	20.7	2.40	22.3	28.0	19.79	18.4	19.0	7.83	7.30	8.7	2.32	21.66	16.6	3.95	36.9	64.2
					2.49			2.43			1.69			0.55			1.78			6.56

Dunnet test	FAS	KI67	MCT8	LAT4	MET	LGALS3
Exp1	p.value.adj	p.value.adj	p.value.adj	p.value.adj	p.value.adj	p.value.adj
1wX-1.5Gy vs C	0.455	0.754	0.991	0.479	0.756	0.972
1wX-3Gy vs C	0.065	0.525	0.556	0.045	0.962	0.304
1wX-6Gy vs C	0.032	1.000	0.713	0.040	0.999	0.763
1wX-12Gy vs C	0.000	0.265	0.045	0.006	0.877	0.015
Exp2						
1wX-12Gy vs C	0.001	0.611	0.043	0.030	0.551	0.627
2wX-12Gy vs C	0.470	0.817	0.821	0.078	0.578	0.998
4wX-12Gy vs C	0.126	0.805	0.046	0.078	0.933	0.076
8wX-12Gy vs C	0.978	0.985	0.966	0.833	0.679	0.046
Exp3						
1wX vs C	0.008	0.189	0.646	0.035	0.221	0.060
IDD vs C	0.001	0.003	0.239	0.009	0.000	0.042
1wX-IDD vs C	0.001	0.003	0.288	0.005	0.001	0.001

Table 3 Serum TT3, TT4 and TSH Data

Exp1		TT3			TT4			TSH		
Control	W221	2.78			5.04			3.48		
	W222	2.67			5.26			3.28		
	W223	3.49			5.75			3.30		
	W224	3.07			5.73			2.94		
	W225	3.81	Mean	SE	4.64	Mean	SE	4.58	Mean	SE
	W226	4.02	3.31	0.23	5.70	5.35	0.19	4.73	3.72	0.31
1wX-1.5Gy	W227	2.42			5.94			4.43		
	W228	3.32			4.87			2.88		
	W229	3.55			7.44			3.57		
	W230	2.38			6.38			3.14		
	W231	2.33			6.57			3.62		
	W232	3.13	2.86	0.22	4.90	6.02	0.41	4.57	3.70	0.28
1wX-3Gy	W233	2.50			4.16			3.48		
	W234	3.57			5.06			4.48		
	W235	3.31			3.53			3.55		
	W236	1.81			5.49			3.60		
	W237	3.28			6.35			3.83		
	W238	3.12	2.93	0.27	6.24	5.14	0.46	4.78	3.95	0.22
1wX-6Gy	W239	2.38			5.46			4.37		
	W240	2.88			4.52			5.66		
	W241	2.66			4.94			4.20		
	W242	1.84			7.27			3.81		
	W243	2.39			4.75			5.40		
	W244	3.08	2.54	0.18	3.58	5.09	0.50	4.66	4.68	0.29
1wX-12Gy	W245	2.80			6.53			6.16		
	W246	3.07			4.78			10.24		
	W247	3.06			3.76			7.19		
	W248	2.02			4.13			5.84		
	W249	3.05			4.23			6.88		
	W250	2.49	2.75	0.17	4.70	4.69	0.40	6.22	7.09	0.66
					0.00					
Exp2										
Control	W251	2.74			5.51			3.70		
	W252	3.02			5.94			4.93		
	W253	2.22			7.38			4.60		
	W254	1.96			7.90			7.51		
	W255	2.01	2.39	0.21	7.21	6.79	0.45	7.45	5.64	0.78
1wX-12Gy	W256	2.05			6.75			7.65		
	W257	1.81			5.15			8.32		
	W258	1.78			6.37			6.40		
	W259	2.53			3.97			5.38		
	W260	2.78	2.19	0.20	3.15	5.08	0.69	5.82	6.71	0.55
2wX-12Gy	W261	1.91			8.99			6.53		
	W262	1.98			6.62			6.54		
	W263	2.34			5.75			7.55		
	W264	1.72	1.99	0.13	5.92	6.82	0.75	7.29	6.98	0.26
4wX-12Gy	W265	1.88			7.06			7.07		
	W266	2.25			6.15			6.09		
	W267	2.14			4.67			5.97		
	W268	2.22			4.36			7.67		
	W269	1.26	1.95	0.19	8.35	6.12	0.74	7.44	6.85	0.35
8wX-12Gy	W270	1.17			7.47			5.40		
	W271	1.76			8.49			7.83		
	W272	1.74						8.78		
	W273	1.96			6.14			7.34		
	W274	2.01	1.73	0.15	7.50	7.40	0.48	8.17	7.51	0.58
Exp3	TT3				TT4			rTSH		
Control	W191	2.22			4.66			3.51		
	W192	2.32			6.44			6.01		
	W193	2.95			6.58			4.52		
	W194	1.60	2.27	0.28	8.17	6.46	0.72	4.94	4.75	0.52
1wX-12Gy	W195	2.43			5.81			6.22		
	W196	1.89			5.59			5.32		
	W197	2.26	2.19	0.16	3.66	5.02	0.68	4.99	5.51	0.37
IDD	W198	2.79			0.44			65.11		
	W199	1.55			0.33			102.14		
	W200	2.05			0.39			71.51		
	W201	2.29			0.38			50.03		
	W202	2.28			0.56			68.15		
	W203	2.55			0.62			62.66		
	W204	2.94	2.35	0.18	0.47	0.46	0.04	80.73	71.47	6.20
1wX-12Gy-IDI	W205	1.49			0.34			128.55		
	W206	2.59			0.35			99.75		
	W207	2.01			0.62			58.89		
	W208	2.87			0.39			106.77		
	W209	2.10			1.30			65.08		
	W210	1.77			0.95			70.56		
	W211	1.73			0.65			61.35		
	W212	1.54	2.01	0.18	1.12	0.72	0.13	67.58	82.32	9.14

Dunnet test	TT3	TT4	TSH
Exp1	p.value.adj	p.value.adj	p.value.adj
1wX-1.5Gy vs C	0.229	0.998	0.830
1wX-3Gy vs C	0.351	0.755	0.570
1wX-6Gy vs C	0.037	0.727	0.072
1wX-12Gy vs C	0.110	0.271	0.004
Exp2			
1wX-12Gy vs C	0.867	0.246	0.545
2wX-12Gy vs C	0.350	1.000	0.340
4wX-12Gy vs C	0.365	0.886	0.409
8wX-12Gy vs C	0.097	0.959	0.201
Exp3			
1wX vs C	0.981	0.334	0.591
IDD vs C	0.984	0.005	0.000
1wX-IDD vs C	0.718	0.007	0.000

Figure 1a and 1b Data

Exp1		colloid area		
Control	W221	3625		
	W222	4077		
	W223	3277		
	W224	2934		
	W225	3655	Mean	SE
	W226	1887	3243	313.4
1wX-1.5Gy	W227	3536		
	W228	2806		
	W229	3730		
	W230	4743		
	W231	2772		
	W232	2182	3295	369.5
1wX-3Gy	W233	1655		
	W234	2736		
	W235	3065		
	W236	3264		
	W237	4097		
	W238	2790	2934	325.3
1wX-6Gy	W239	669		
	W240	1549		
	W241	2674		
	W242	1905		
	W243	2976		
	W244	1926	1950	335.8
1wX-12Gy	W245	2993		
	W246	1502		
	W247	728		
	W248	910		
	W249	1207		
	W250	933	1379	341.1
Exp2				
Control	W251	5411		
	W252	3922		
	W253	2744		
	W254	3104		
	W255	1830	3402	604.1
1wX-12Gy	W256	413		
	W257	468		
	W258	509		
	W259	484		
	W260	747	524	57.8
2wX-12Gy	W261	2975		
	W262	2905		
	W263	4524		
	W264	4696	3775	483.5
4wX-12Gy	W265	3035		
	W266	4460		
	W267	3449		
	W268	2223		
	W269	1855	3004	460.8
8wX-12Gy	W270	2277		
	W271	3965		
	W272	3846		
	W273	4040		
	W274	3411	3508	326.3

Dunnet test

Exp1	p.value.adj
1wX-1.5Gy vs C	0.846
1wX-3Gy vs C	0.541
1wX-6Gy vs C	0.032
1wX-12Gy vs C	0.005
Exp2	
1wX-12Gy	0.020
2wX-12Gy	0.928
4wX-12Gy	0.910
8wX-12Gy	0.999

Figure 2a, 2b, 2c, and 2d & Figure 4a and 4b Data

Exp1		TUNEL			Ki67		
Control	W221	3.00			17.17		
	W222	1.14			25.00		
	W223	0.50			13.00		
	W224	1.00			18.00		
	W225	0.83	Mean	SE	15.80	Mean	SE
	W226	1.57	1.34	0.36	30.50	19.9	2.67
1wX-1.5Gy	W227	0.75			32.00		
	W228	1.29			27.67		
	W229	0.14			22.50		
	W230	1.00			7.83		
	W231	0.33			12.00		
	W232	1.17	0.78	0.19		20.4	4.58
1wX-3Gy	W233	0.71			27.17		
	W234	1.00			12.00		
	W235	0.33			16.83		
	W236	0.17			18.00		
	W237	1.00			14.60		
	W238	1.83	0.84	0.24	23.83	18.7	2.34
1wX-6Gy	W239	2.29			35.67		
	W240	1.57			28.50		
	W241	1.38			13.83		
	W242	0.36			13.50		
	W243	2.00			14.67		
	W244	1.38	1.50	0.27	17.67	20.6	3.78
1wX-12Gy	W245	1.00			18.50		
	W246	2.67			40.00		
	W247	4.40			28.60		
	W248	4.00			55.83		
	W249	4.20			26.83		
	W250	2.63	3.15	0.53	15.67	30.9	6.09
Exp2							
Control	W251	0.00			5.17		
	W252	1.00			9.14		
	W253	1.00			6.80		
	W254	0.25			6.57		
	W255	0.40	0.53	0.20	9.25	7.4	0.79
1wX-12Gy	W256	2.83			3.17		
	W257	3.00			12.38		
	W258	4.33			16.00		
	W259	4.67			14.00		
	W260	4.00	3.77	0.36	17.86	12.7	2.55
2wX-12Gy	W261	1.33			11.83		
	W262	0.50			4.17		
	W263	1.00			5.75		
	W264	0.25	0.77	0.24	2.67	6.1	2.01
4wX-12Gy	W265	0.80			8.00		
	W266	0.40			24.57		
	W267	1.40			7.88		
	W268	0.80			6.13		
	W269	2.14	1.11	0.30	10.25	11.4	3.37
8wX-12Gy	W270	0.50			8.86		
	W271	0.29			6.00		
	W272	0.40			10.33		
	W273	0.00			8.43		
	W274	2.83	0.80	0.51	25.40	11.8	3.47
Exp3	TT3						
Control	W191	3.67			17.33		
	W192	0.40			12.33		
	W193	0.00			13.83		
	W194	0.17	1.06	0.87	9.71	13.3	1.59
1wX-12Gy	W195	6.67			14.43		
	W196	3.88			11.00		
	W197	3.33	4.63	1.03	11.14	12.2	1.12
IDD	W198	1.83			83.43		
	W199	7.00			52.00		
	W200	41.63			76.00		
	W201	19.50			48.57		
	W202	13.20			54.88		
	W203	5.50			50.63		
	W204	5.71	13.48	5.19	50.25	59.4	5.36
1wX-12Gy-IDI	W205	6.75			40.20		
	W206	3.00			32.29		
	W207				21.20		
	W208	3.50			32.00		
	W209						
	W210						
	W211				14.60		
	W212	2.83	4.02	0.92	11.80	25.3	4.58
1wX-12Gy-IDI	W205	9.80			153.75		
	W206 no						
	W207	6.20			191.75		
	W208 no						
	W209	6.78			141.20		
	W210	13.80			52.67		
	W211	4.40			53.83		
	W212	5.17	7.69	1.44	91.00	114.03	23.29

Dunnet test	TUNEL	Ki-67
Exp1	p.value.adj	p.value.adj
1wX-1.5Gy vs C	0.982	1.000
1wX-3Gy vs C	0.973	0.993
1wX-6Gy vs C	0.638	1.000
1wX-12Gy vs C	0.032	0.399
Exp2		
1wX-12Gy	0.001	0.325
2wX-12Gy	0.888	0.948
4wX-12Gy	0.439	0.701
8wX-12Gy	0.891	0.652
Exp3		
1wX	0.034	0.991
IDD	0.092	0.001
1wX-IDD(Hyper)	0.072	0.090
1wX-IDD(AD)	0.009	0.033
